# Optimal Decision-Making of Closed-Loop Supply Chains in E-Commerce Platform Considering Sales Cooperations under Environmental Effects and WEEE Regulations

**DOI:** 10.3390/ijerph20095724

**Published:** 2023-05-04

**Authors:** Manyi Tan, Fei Pei, Li He, Hong Cheng, Shupeng Huang

**Affiliations:** 1 College of Management Science, Chengdu University of Technology, Chengdu 610059, China; tanmy@cdut.edu.cn (M.T.);; 2 Southwest Jiaotong University Hope College, Chengdu 610400, China

**Keywords:** WEEE regulation, e-commerce platform, sales cooperation mode, environmental effect, e-commerce closed-loop supply chain (ECLSC)

## Abstract

Nowadays, to achieve carbon neutrality, e-commerce platforms participate in the sales and recycling of electrical and electronic products in consideration of waste electrical and electronic equipment (WEEE) regulations and environmental effects. This study builds a Stackelberg game model for an e-commerce closed-loop supply chain (ECLSC) under different sales cooperation modes between a manufacturer of electrical and electronic products and an e-commerce platform. Reverse induction is used to obtain the optimal decision-making and profit of the ECLSC under three sales cooperation modes, considering the influence of environmental effects on optimal decision and objective functions. The results show the following: the sales cooperation mode and environmental cost do not affect the WEEE recovery prices of manufacturers and e-commerce platforms, nor do they affect government subsidy standards for dismantling WEEEs; they are, however, positively correlated with environmental benefits. Furthermore, the wholesale and retail prices of electrical and electronic products under different sales cooperation modes are related to sales cooperation modes and environmental costs. Moreover, the processing fees imposed on the manufacturers are related to the environmental costs of the electrical and electronic products; the thresholds of environmental costs of products for government to levy processing fees are different under different sales cooperation modes. Finally, the environmental cost of products required by the government’s levying of processing fees are the lowest under a hybrid model. Generally speaking, under WEEE regulations, governments should levy more processing fees for electrical and electronic products with higher environmental costs. Meanwhile, increased environmental benefits will always increase the profits of supply chain members, but increased environmental costs do not always reduce the profits of supply chain members, and multichannel product sales do not always generate profits for manufacturers.

## 1. Introduction

Manufacturing is important to a country’s comprehensive national power. After the internet bubble and the global financial crisis, many countries, especially developed nations, began to recognize the importance of the manufacturing industry. The emergence of the Fourth Industrial Revolution has compelled many countries to develop corresponding strategies related to manufacturing, such as the National Network for Manufacturing Innovation in the US, Industry 4.0 in Germany, the Industrial Value Chain Initiative in Japan, and Made in China 2025 in China. In this new era, intelligent manufacturing and technological progress have promoted electrical and electronic product development and decreased product costs. Despite such progress, the amount of global electrical and electronic waste reached 53.6 million tons in 2020 and can rise to 74.7 million tons by 2030 [1]. Therefore, to deal with such a huge amount of e-waste, appropriate recycling mechanisms should be designed and implemented. Otherwise, the inappropriate e-waste treatment can introduce harmful substances into the environment.

In consideration of such environmental challenges, in 2002, the European Union took the lead in issuing waste electrical and electronic equipment (WEEE) regulations to clarify producer responsibilities and product identification requirements [2]. Later, in 2011, China began to implement regulations for WEEE recycling and developed measures for the collection and use of fees [3]. According to these measures, WEEE processing fees are collected according to the quantity quota of electrical and electronic products sold by producers or imported by consignees or agents, and a fixed amount of subsidy is given to processing enterprises according to the actual number of disassembled WEEEs. In this context, modern manufacturing enterprises face severe external constraints and are therefore compelled to adopt measures to achieve green development.

Meanwhile, the rapid development of the internet has had profound effects on all walks of life. At the 2018 Davos Forum, Ma Yun, cofounder and former chairman of the Alibaba Group, proposed that “e-commerce must be the future”. Under this trend, it has become an inevitable choice for enterprises to open online sales channels to obtain higher profits. Enterprises usually do this in one of two ways: they build their own platform or cooperate with third-party platforms. The advantages of self-built platforms include autonomy, high flexibility, personalized service, and no commission pressures. There are, however, a number of disadvantages. First, platform construction and maintenance costs are high, which is especially challenging for small and medium-sized enterprises. Second, there are high technical requirements and publicity costs. Therefore, self-built platforms are typically more suitable for large enterprises in China, such as Huawei, Midea, and Lenovo. For small and medium-sized enterprises, cooperation with third-party platform enterprises is usually the optimal choice. The development of such online sales channels not only expands sales channels, but is also good for recycling. As such, online channels can play an important role in WEEE recycling and resource utilization.

From the perspective of manufacturers (suppliers), there are three typical modes of sales cooperation with third-party e-commerce platforms: direct sales, wholesale sales, and hybrid sales.

In the direct sales mode, the manufacturer enters an e-commerce platform and opens its own store; it directly sells goods through the e-commerce platform and pays a commission to the platform. The platform only collects the commission and provides the corresponding services; it does not participate in commodity transactions between consumers and manufacturers. Examples include Alibaba’s 1688 (www.1688.com) and Yiwugo.com.

In the wholesale sales mode, the manufacturer wholesales products to an e-commerce platform. The e-commerce platform then sells the products and provides a unified one-stop service that includes warehousing, logistics, and platform operations. Examples include Vipshop and Best Buy.

In the hybrid sales mode, the manufacturer also wholesales products to an e-commerce platform, which then sells the products. In addition, the manufacturer directly enters the e-commerce platform and opens its own store, directly sells goods through the platform, and pays a commission. Typical hybrid platforms include Amazon, Jingdong, Tmall, Cdiscount, and Newegg.

In addition to product sales, e-commerce platforms have introduced online recycling. With the development of e-commerce, online recycling has also developed rapidly. For example, Jingdong, Amazon, and other e-commerce platforms all provide online recycling businesses.

In light of the above, this study mainly takes the manufacturer’s (supplier’s) decisions into account in an e-commerce closed-loop supply chain (ECLSC) and considers mathematical models under the following assumption. First, we assume the government will levy processing fees for producing each unit of electrical and electronic products because of the environmental harmfulness generated during their production from the manufacturer. Second, we consider the environmental effects of the electrical and electronic products which include the environmental cost of consuming them (e.g., pollution) and the environmental benefit of recycling the used products (e.g., pollution reduction). Third, we holistically consider the subsidies which are the government subsidies provided for recycling, and the social welfare and consumer surpluses which are the total benefits obtained by society from the operations of the ECLSC and surplus by consumers from purchasing the products. Specifically, we address the following questions:

(1) How can a government design appropriate policies for levying processing fees on the production of electrical and electronic products, as well as for providing subsidies for dismantling WEEEs under environmental effects and different cooperation modes?
(2) How can environmental effects and different cooperation modes influence the decision making of ECLSC members, including their pricing decisions for selling products and recycling?
(3) How do the environmental effects of products (i.e., environmental cost and benefit) influence the profits of ECLSC members and society and consumer welfare under different cooperation modes?

The rest of this article is organized as follows. Section 2 reviews the literature and clarifies the study’s contributions. The problem formulation and model hypothesis are described in Section 3. Section 4 analyzes ECLSC under the three sales cooperation modes, while Section 5 compares and analyzes the ECLSC’s optimal decision-making under the three cooperation modes. The numerical analysis is presented in Section 6, while Section 7 concludes the research.

## 2. Literature Review

This section reviews the related literature in terms of the following aspects: closed-loop supply chain and ECLSC, supply chain cooperation mode, supply chain management under government regulation, and the effect of environmental factors on supply chains.

Many studies of closed-loop supply chains have focused on channel or pricing decision-making. Savaskan et al. [4] established a decentralized decision model for a closed-loop supply chain with three recycling channels: manufacturer recycling, retailer recycling, and third-party recycling. Choi et al. [5] analyzed the optimal decision-making of supply chain members under three dominant modes: manufacturer as leader, retailer as leader, and recycler as leader. Studying the influence of the channel power structure on the optimal decision-making and profit of a closed-loop supply chain, Gao et al. [6] found that the power transfer from manufacturer to retailer always increases the retailer’s profit. Huang and Wang [7] developed three hybrid closed-loop supply chain models involving recycling and remanufacturing operations among supply chain members. On that basis, the study discussed the effects of remanufacturing capability on supply chain members and the environment. Giri et al. [8] studied a closed-loop supply chain including a positive dual channel (traditional retail channel and e-retail channel) and reverse dual channel (third-party channel and e-retail channel), and the e-retail channel simultaneously works as the main body of sales and recycling. Constructing a dual-channel closed-loop supply chain including manufacturers, retailers, and online recycling platforms, Zhu et al. [9] found that consumer bargaining behavior affects the direct recovery price of online recycling platforms. Against the background of O2O, Kong et al. [10] established a benchmark model for dual-channel price and service competition comprising an e-channel and a traditional channel; on that basis, they discussed the solutions for channel conflict and double marginalization in a closed-loop dual-channel distribution network. Duan et al. [11] established a multiperiod ECLSC model using variational inequality and analyzed the relationship between consumer preferences and supply chain members’ profits. Tang et al. [12] established a closed-loop supply chain composed of manufacturers and retailers regarding warranties for new and remanufactured products and compared the equilibrium decision-making, profit margin, and consumer surplus of manufacturer and retailer warranties. He et al. [13] developed the model of competitive collection in a closed-loop supply chain where a manufacturer and a third-party logistics provider involve the recycling operations. They found that the competitive collection is highly influenced by the channel discrimination and collection convenience.

The abovementioned research on closed-loop supply chains has mainly focused on supply chain structure, channel selection, and decision-making changes among supply chain members. Although some studies have raised the issue of sales and recycling through online channels, none of them have considered the cooperation mode between manufacturers and online platforms.

Regarding research on supply chain cooperation modes, Ma et al. [14] studied the influence of different cooperation strategies on decision-making in a three-level closed-loop supply chain composed of a manufacturer, two recyclers, and one retailer. Li et al. [15] studied a cooperative reverse supply chain model composed of a single recycler, a single remanufacturer, and two retailers under the condition of complete information; this study further considered the influence of the market demand for remanufactured products and the market share of two retailers on self-owned decisions. Under the condition of uneconomical manufacturer scale, Shi and Nie [16] considered four cooperation strategies: no cooperation between manufacturer and supply chain members, cooperation between manufacturer and retailer, cooperation between manufacturer and recycler, and cooperation between manufacturer and supply chain members. In this way, they aimed to obtain each member’s optimal decisions regarding wholesale price, sales price, recovery rate, and profit. Zheng et al. [17] used a cooperative game method to study a closed-loop supply chain with four alliance modes in the presence of fairness-concern behavior. Establishing a closed-loop supply chain model composed of manufacturers, retailers, and network service platforms, Xiang and Xu [18] found that wholesale and sales prices are higher when manufacturers cooperate with network service platforms. Xie et al. [19] built an evolutionary game model for a closed-loop supply chain to investigate the cooperations between manufacturers and e-commerce platform, and they found the incentives to join the cooperations depend on the cost of building the recycling system, which can be affected by the government interventions.

Most of the abovementioned studies focused on cooperation between manufacturers and different supply chain members but ignored online platforms. Although Xiang and Xu [18] and Xie [19] examined cooperation between manufacturers and platforms, the mode of cooperation they considered is relatively simple, meaning that they did not fully consider the effect of different sales cooperation modes between manufacturers and e-commerce platforms from a perspective of closed-loop supply chains.

Many studies have investigated the effects of government regulations on supply chains. Hammond and Beullens [20] examined closed-loop supply chain network equilibrium problems between electrical and electronic product manufacturers and consumers under WEEE regulations. Chen and Sheu [21] found that environmental regulation pricing strategies can promote the extended product responsibility of enterprises in green supply chains in a competitive market. Analyzing the effect of government consumption subsidies on closed-loop supply chains and consumers, Ma et al. [22] found that consumption subsidies can benefit consumers, manufacturers, and retailers, and can expand closed-loop supply chain revenues. He et al. [23] studied the channel structure of dual-channel closed-loop supply chains and obtained optimal government subsidy levels for different channel structures; higher subsidy levels were found to always benefit consumers, as well as the whole supply chain. Tan and Guo [24] found that trading market efficiency can only be ensured if factors such as recycling rewards, recycling penalties, and remanufacturing technology subsidies are within a certain range. Bai et al. [25] studied two situations where manufacturers recycle themselves or manufacturers outsource recycling services to third-party platforms with and without government subsidies to obtain the best price and service quality strategies, respectively. These studies of government regulation generally lack the application of the consideration of collecting and processing fees for new products and subsidies for remanufactured production to the supply chain, especially ECLSC.

In addition, the influence of environmental factors on supply chains has received considerable attention. Georgiadis and Besiou [26] studied the environmental sustainability strategies and operational characteristics of closed-loop supply chains using system dynamics and analyzed their effects on closed-loop WEEE supply chains. Fahimnia et al. [27] evaluated the environmental effects of forward and reverse supply chains by analyzing their functional costs. Toptal and Cetinkaya [28] studied the effect of supply chain coordination on the environment through the carbon footprint. Sarkar et al. [29] considered the environmental effects of returnable transportation projects, remanufacturing, and transportation and analyzed the effect on remanufacturing by adopting transportation costs and carbon emission costs. Wang et al. [30] found that mandatory remanufacturing does not necessarily bring about better environmental effects but may, in fact, have serious environmental consequences. Liu et al. [31] discussed the effect of product design on a two-level closed-loop supply chain. Through environmental effect analysis, the cost range beneficial to remanufacturing activities was determined, and the conditions for environmentally friendly product design were also obtained. Hasan et al. [32] investigated the inventory decisions, as well as the investment of technology under the different types of environmental regulations on the carbon emission of the supply chain. However, these studies have rarely adopted environmental costs and benefits to analyze the benefits of supply chain members, government social welfare, and consumer surplus, especially in the context of ECLSC.

The present study focuses on manufacturers selling products and recycling WEEEs through online channels, introduces WEEE government regulations (processing fee policies for producing products and WEEE dismantling subsidy policies), studies different sales cooperation modes between manufacturers and e-commerce platforms under ECLSC, and analyzes the effect of environmental cost and environmental benefit changes on ECLSC.

## 3. Problem Description and Model Assumption

### 3.1. Problem Description

Here, we consider an ECLSC composed of manufacturers, e-commerce platforms, and consumers. The manufacturer is responsible for producing new electrical and electronic products and reproducing remanufactured products (there is no difference in quality and price between new products and remanufactured products [4]) and then selling new and remanufactured products through an e-commerce platform. The e-commerce platform provides an online sales platform selling new and remanufactured products, and it recycles WEEEs. The government levies processing fees for production according to the actual number of electrical and electronic products produced and gives quota subsidies to qualified processors according to the actual number of WEEEs.

Considering WEEE government regulations, according to different sales cooperation modes between manufacturers and e-commerce platforms, this study establishes three ECLSC models with manufacturers as the core.

**Figure 1:**
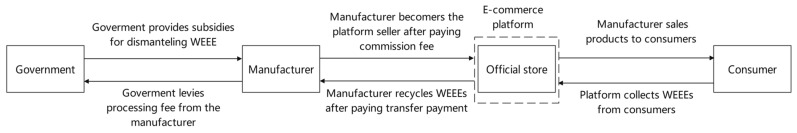
ECLSC mode for direct sales (T mode).

**Figure 2:**
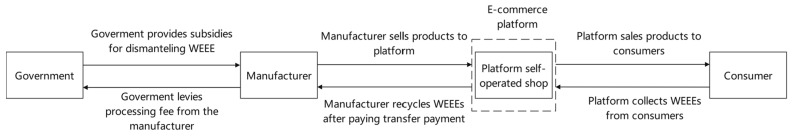
ECLSC mode for wholesale sales (Z mode).

**Figure 3:**
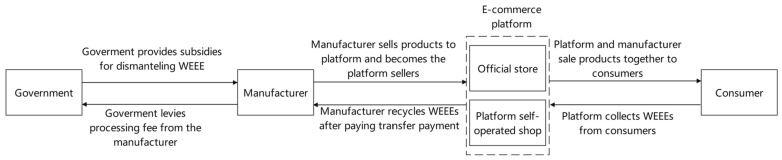
ECLSC mode for hybrid sales (H mode).

(1) ECLSC mode for direct sales (T mode) (Figure 1). In this model, the e-commerce platform is a pure platform. It charges the manufacturer’s commission, allows the manufacturer to sell electrical and electronic products through the platform, and recycles WEEEs from consumers to sell to the manufacturer to earn profits. The manufacturer obtains the qualification to deal with WEEEs from the government and pays the platform a commission to sell directly to consumers through the platform. Meanwhile, the manufacturer purchases WEEEs from the platform at a certain price for disassembly and remanufacturing. After remanufacturing, the remanufactured products are also sold through this platform. The government levies WEEE processing fees according to the actual number of electrical and electronic products and gives a quota subsidy to the manufacturer according to the actual number of WEEEs.

(2) ECLSC wholesale mode (Z mode) (Figure 2). In this mode, the manufacturer wholesales electrical and electronic products to the e-commerce platform. The platform sells the products directly to consumers through online channels, conducts online WEEE recycling activities, and resells recycled WEEEs to the manufacturer with WEEE processing qualification approved by the government. The manufacturer disassembles WEEEs and remanufactures products. The government levies WEEE processing fees according to the actual number of electrical and electronic products and gives quota subsidies to the manufacturer according to the actual number of WEEEs.

(3) ECLSC mode of hybrid sales (H mode) (Figure 3). In this mode, on the one hand, the manufacturer wholesales electrical and electronic products to the e-commerce platform; on the other hand, the manufacturer opens a direct store on the platform and directly sells products to consumers (at this time, the manufacturer must pay a commission to the e-commerce platform operator). The platform charges commissions from the manufacturer, sells products through a self-operated platform, and conducts online WEEE recycling to resell recycled WEEEs to the manufacturer.

### 3.2. Model Assumptions

(1) The manufacturer adopts new materials or materials obtained after disassembling WEEE to produce electrical and electronic products. There is no difference in quality between the electrical and electronic products produced by the two materials, and the prices are the same. $c_{n}$ is the unit cost of the manufacturer using new materials to produce new products, $c_{r}$ is the unit cost of remanufacturing using obtained old materials,$\;c_{d}$ is the unit cost of the manufacturer’s disassembly, $B^{j}$ is the transfer payment price of WEEE recycled from e-commerce platform, and $c_{n}-c_{r}-B^{j}-c_{d}>0$. This means the manufacturer can save costs by remanufacturing, and remanufacturing activities are profitable.

(2) Assuming that demand is linear, the demand function is $D_{i}^{j}=\alpha -{\beta P}_{i}^{j}$. The demand functions under the direct sales (T mode) and wholesale (Z mode) ECLSC models are $D_{M}^{T}=\alpha -\beta P_{M}^{T}$ and $D_{E}^{Z}=\alpha -\beta P_{E}^{Z}$, respectively. For the hybrid sales (H mode) ECLSC model, electrical and electronic products can be sold through two channels: the manufacturer’s direct sales channel and the e-commerce platform’s self-operated channel. Consumers no longer choose products based solely on the price of a single channel; instead, they compare the price of the same product in both channels and then decide which channel to buy from. Referring to the demand function design method in Park and Keh [33] and Ma et al. [34], the sensitivity coefficient $\beta_{1}$ of consumers to the price difference between the manufacturer’s direct sales channel and self-operated platform channel is introduced. At this time, the manufacturer’s direct sales price is $P_{M}^{H}$, the platform self-operated price is $P_{E}^{H}$, and $P_{M}^{H}\geq P_{E}^{H}$. Market capacity is determined by the manufacturer’s direct sales price, and the self-operated platform channel will not change the total market capacity but will occupy the demand of the original direct sales channel. Thus, the demand of the manufacturer’s direct sales channel can be set as

$$D_{M}^{H}=\alpha -\beta P_{M}^{H}-\beta_{1}\left(P_{M}^{H}-P_{E}^{H}\right)$$

The demand of the self-operated platform channel is

$$D_{E}^{H}=\beta_{1}\left(P_{M}^{H}-P_{E}^{H}\right)$$

(3) Assuming that WEEE recovery from the e-commerce platform is $Q^{j}$, and $Q^{j}=Q_{0}+\delta A^{j}$, where δ>0.

(4) Assuming the manufacturer disassembles the WEEEs, and all recycled materials can be used for remanufacturing.

(5) According to the policy, the government levies a fixed fee amount $h^{j}$ for each unit of production and, at the same time, a fixed subsidy amount $g^{j}$ for each unit of WEEE recycling.

(6) Referring to Ma et al. [34], it is assumed that the environmental cost generated by consuming each unit of electrical and electronic products is C, while the environmental benefit generated by recycling each unit of WEEE is V.

(7) The cost of WEEE dismantling by the manufacturer is $c_{d};\;c_{n}-c_{r}+g-B^{j}-c_{d}>0$ indicates that it is profitable for the manufacturer to disassemble. $V+c_{n}>S_{0}+c_{d}+c_{r}$ indicates that the recycling, dismantling, and remanufacturing process is beneficial to the environment.

(8) Assuming all members of the ECLSC make their own decisions under the condition of complete information.

(9) To ensure the significance of the research problem, it is assumed that the model parameters meet the following requirements:

$$\beta_{1}>2\beta ,\;\frac{\alpha -\beta c_{n}}{2\beta }<C<\frac{\alpha -\beta c_{n}}{\beta }$$

## 4. Analysis of ECLSC Model under Three Sales Cooperation Modes

Different sales cooperation modes between the manufacturer and e-commerce platform form different ECLSCs. Considering government regulation, a three-stage Stackelberg game ECLSC model is constructed. The reason why Stackelberg game is applied in this paper is because the players (i.e., government, manufacturer, and platform) in the game are not equally powerful. Instead, due to the difference of the leadership in each player, the moves are sequential among difference players, with the move first from the government, then the manufacturer and finally the platform. Therefore, to clearly depict and investigate such a sequence in the game, Stackelberg game is chosen as the appropriate method. The decision-making sequence of the supply chain members is as follows.

The first stage is the formulation of government policies. With the goal of maximizing social welfare, the government determines the standards for levying processing fees for electrical and electronic product units and unit subsidies for dismantling and disposing of WEEEs.

The second stage is the manufacturer’s decision. According to policies and the principle of maximizing its own profits, the manufacturer determines the wholesale price or direct price according to different sales cooperation modes with the platform (the commission rate of the platform is fixed when the manufacturer opens a store for direct sales on the platform, which is an exogenous variable) and the price of recycling waste from the e-commerce platform.

The third stage is the e-commerce platform’s decision-making. Based on the principle of maximizing its own profits, the platform sets the retail price for self-sales of products and determines the unit price for recycling waste from consumers.

The ECLSC model under different sales cooperation modes can be solved by reverse induction.

### 4.1. ECLSC Mode for Direct Sales (T Mode)

Under this condition, the manufacturer sets up direct-sales flagship stores on the e-commerce platform. The decision variables include the direct sales price $P_{M}^{T}$ and the price $B^{T}$ of recycling waste from the e-commerce platform. The decision variable of the e-commerce platform is the recycling price $A^{T}$ from consumers. The objective functions of the e-commerce platform, the manufacturer, and the government are, respectively,

(1) $$\prod_{E}^{T}=\rho P_{M}^{T}D_{M}^{T}+(B^{T}-A^{T})Q^{T}-S_{0}Q^{T}-SD_{M}^{T}$$

(2) $$\prod_{M}^{T}=(P_{M}^{T}-c_{n})D_{M}^{T}+(\Delta +g^{T}-B^{T}-c_{d})Q^{T}-h^{T}D_{M}^{T}-\rho P_{M}^{T}D_{M}^{T}$$

(3) $$\prod_{G}^{T}=\prod_{M}^{T}+\prod_{E}^{T}+X^{T}+h^{T}D_{M}^{T}-g^{T}Q^{T}-CD_{M}^{T}+VQ^{T}$$

where $\Pi_{E}^{T}$ represents the profit of the e-commerce platform. The first item is the commission income from the manufacturer renting the platform, the second is the income from recycling WEEEs, the third is the cost paid by WEEEs, and the fourth is the operational cost of the e-commerce platform. $\Pi_{M}^{T}$ represents the manufacturer’s profit. The first item is the profit from product sales, the second is the income from recycling WEEEs (including government subsidies) and the costs saved in the remanufacturing process, the third is the processing fee collected by the government, and the fourth is the commission paid to the e-commerce platform. $\Pi_{G}^{T}$ represents the government’s benefits (social welfare). The first item is the manufacturer’s profits, the second is the e-commerce platform’s profits, the third is consumer surplus, the fourth is the processing fee collected by the government, the fifth is the government’s subsidy for dismantling and processing WEEEs, the sixth is the environmental cost of consumer electrical and electronic products, and the seventh is the environmental benefit brought by recycling WEEEs.

Consumer surplus is the sum of consumer surplus in the forward supply chain and the reverse supply chain, namely,

(4) $$X^{T}=\frac{\alpha^{2}}{2\beta }-\alpha P_{M}^{T}+\frac{\beta {\left(P_{M}^{T}\right)}^{2}}{2}+Q_{0}A^{T}+\frac{\delta {\left(A^{T}\right)}^{2}}{2}$$

All of the calculations of consumer surpluses are in Appendix A.

According to the reverse-induction method, the optimal decisions of the ECLSC members in the T mode can be obtained:

$$g^{T^{*}}=\frac{(4V-3S_{0}-3c_{d}+3c_{n}-3c_{r})\delta +3Q_{0}}{\delta }$$

$$h^{T^{*}}=\frac{2(C+c_{n}+S)(1-\rho )\beta -c_{n}\beta +\alpha (\rho -1)}{\beta }$$

$$P_{M}^{T^{*}}=C+c_{n}+S$$

$$A^{T^{*}}=V-S_{0}+c_{n}-c_{d}-c_{r}$$

$$B^{T^{*}}=\frac{(2V-S_{0}+2c_{n}-2c_{d}-2c_{r})\delta +Q_{0}}{\delta }$$

At this point, the manufacturer’s optimal profit is

$$\begin{array}{l} \prod_{M}^{T^{*}}=\frac{1}{\delta \beta }(2\beta {\left(V-S_{0}+c_{n}-c_{d}-c_{r}\right)}^{2}\delta^{2}+(-{\left(C+S+c_{n}\right)}^{2}(\rho -1)\beta^{2}\\ +(2(\alpha (\rho -1)+2Q_{0})c_{n}+2\alpha (C+S)(\rho -1)+4Q_{0}(V-c_{r}-S_{0}-c_{d}))\beta \\ -\alpha^{2}(\rho -1))\delta +2\beta Q_{0}{}^{2}) \end{array}$$

The optimal profit of the e-commerce platform is

$$\begin{array}{l} \prod_{E}^{T^{*}}=\frac{1}{\delta }({\left(V-S_{0}+c_{n}-c_{d}-c_{r}\right)}^{2}\delta^{2}+(-\rho \beta c_{n}{}^{2}+(2Q_{0}+(\alpha -2(C+S)\beta )\rho +S\beta )c_{n}\\ +((C+S)\rho -S)(\alpha -\beta (C+S))+2Q_{0}(V-c_{r}-S_{0}-c_{d}))\delta +Q_{0}^{2}) \end{array}$$

The government’s optimal social welfare is

$$\begin{array}{l} \prod_{G}^{T^{*}}=\frac{1}{2\beta }({\left(C+S+c_{n}\right)}^{2}\beta^{2}+({\left(c_{r}-V+S_{0}+c_{d}-c_{n}\right)}^{2}\delta +2c_{n}(Q_{0}-\alpha )\\ +2Q_{0}(V-c_{d}-c_{r}-S_{0})-2\alpha (C+s))\beta +\alpha^{2}) \end{array}$$

Consumer surplus is

$$\begin{array}{l} X^{T^{*}}=\frac{1}{2\beta }({\left(C+S+c_{n}\right)}^{2}\beta^{2}+({\left(c_{r}-V+S_{0}+c_{d}-c_{n}\right)}^{2}\delta +2c_{n}(Q_{0}-\alpha )\\ +2Q_{0}(V-c_{d}-c_{r}-S_{0})-2\alpha (C+s))\beta +\alpha^{2}) \end{array}$$

### 4.2. ECLSC Mode for Wholesale Sales (Z Mode)

Here, the manufacturer’s decision variables are the wholesale price $w^{z}$ of the product and the price $B^{Z}$ of WEEEs recovered from the e-commerce platform. The e-commerce platform’s decision variables are the recycling price $A^{Z}$ from consumers and the self-sold retail price $P_{E}^{Z}$.

The objective functions of the e-commerce platform, the manufacturer, and the government are as follows:

(5) $$\prod_{E}^{Z}=(P_{E}^{Z}-w^{Z})D_{E}^{Z}+(B^{Z}-A^{Z})Q^{Z}-S_{0}Q^{Z}$$

(6) $$\prod_{M}^{Z}=(w^{Z}-c_{n})D_{E}^{Z}+(\Delta +g^{Z}-B^{Z}-c_{d})Q^{Z}-h^{Z}D_{E}^{Z}$$

(7) $$\prod_{G}^{Z}=\prod_{M}^{Z}+\prod_{E}^{Z}+X^{Z}+h^{Z}D_{E}^{Z}-g^{Z}Q^{Z}-CD_{M}^{Z}+VQ^{Z}$$

where $\Pi_{E}^{Z}$ represents the profit of the e-commerce platform. The first item is the self-operated sales profit of the e-commerce platform, the second is the profit from recycling WEEEs, and the third is the cost paid by WEEEs. $\Pi_{M}^{Z}$ represents the manufacturer’s profit. The first item is the profit from wholesale products to the e-commerce platform, the second is the income from recycling and processing WEEEs (equal to government subsidies minus the cost of recycling WEEEs from the e-commerce platform and then minus the cost of dismantling and processing WEEEs) and cost savings for remanufacturing, and the third is the processing fee levied by the government. $\Pi_{G}^{Z}$ represents the government’s benefits (social welfare). The first item is the manufacturer’s profits, the second is e-commerce platform profits, the third is consumer surplus, the fourth is the processing fee collected by the government, the fifth is the government’s subsidy for dismantling and processing WEEEs, the sixth is the environmental cost of consumer electrical and electronic products, and the seventh is the environmental benefit brought by recycling WEEEs.

Consumer surplus is the sum of consumer surplus in the forward supply chain and the reverse supply chain, namely,

(8) $$X^{Z}=\frac{\alpha^{2}}{2\beta }-\alpha P_{E}^{Z}+\frac{\beta {\left(P_{E}^{Z}\right)}^{2}}{2}+Q_{0}A^{Z}+\frac{\delta {\left(A^{Z}\right)}^{2}}{2}$$

According to the reverse-induction method, the optimal decision of the ECLSC members in the Z mode can be obtained:

$$g^{Z^{*}}=\frac{(4V-3S_{0}-3c_{d}+3c_{n}-3c_{r})\delta +3Q_{0}}{\delta }$$

$$h^{Z^{*}}=\frac{(4C+3c_{n})\beta -3\alpha }{\beta }$$

$$P_{E}^{T^{*}}=C+c_{n}$$

$$A^{Z^{*}}=V-S_{0}+c_{n}-c_{d}-c_{r}$$

$$B^{Z^{*}}=\frac{(2V-S_{0}+2c_{n}-2c_{d}-2c_{r})\delta +Q_{0}}{\delta }$$

$$w^{Z^{*}}=\frac{2(C+c_{n})\beta -\alpha }{\beta }$$

At this point, the manufacturer’s optimal profit is

$$\begin{array}{l} \prod_{M}^{Z^{*}}=\frac{1}{\delta \beta }(2\beta {\left(V-S_{0}+c_{n}-c_{d}-c_{r}\right)}^{2}\delta^{2}+(2{\left(C+c_{n}\right)}^{2}\beta^{2}\\ +(4c_{n}(Q_{0}-\alpha )-4Q_{0}(c_{r}-V+S_{0}+c_{d})-4\alpha C)\beta +2\alpha^{2})\delta +2\beta Q_{0}{}^{2}) \end{array}$$

The optimal profit of the e-commerce platform is

$$\begin{array}{l} \prod_{E}^{Z^{*}}=\frac{1}{\delta \beta }(\beta {\left(V-S_{0}+c_{n}-c_{d}-c_{r}\right)}^{2}\delta^{2}+({\left(C+c_{n}\right)}^{2}\beta^{2}\\ +(2c_{n}(Q_{0}-\alpha )-2Q_{0}(c_{r}-V+S_{0}+c_{d})-2\alpha C)\beta +\alpha^{2})\delta +\beta Q_{0}{}^{2}) \end{array}$$

The government’s optimal social welfare is

$$\begin{array}{l} \prod_{G}^{Z^{*}}=\frac{1}{2\beta }({\left(C+c_{n}\right)}^{2}\beta^{2}+({\left(c_{r}-V+S_{0}+c_{d}-c_{n}\right)}^{2}\delta +2c_{n}(Q_{0}-\alpha )\\ +2Q_{0}(V-c_{d}-c_{r}-S_{0})-2\alpha C)\beta +\alpha^{2}) \end{array}$$

Consumer surplus is

$$\begin{array}{l} X^{Z^{*}}=\frac{1}{2\beta }({\left(C+c_{n}\right)}^{2}\beta^{2}+({\left(c_{r}-V+S_{0}+c_{d}-c_{n}\right)}^{2}\delta +2c_{n}(Q_{0}-\alpha )\\ +2Q_{0}(V-c_{d}-c_{r}-S_{0})-2\alpha C)\beta +\alpha^{2}) \end{array}$$

### 4.3. ECLSC Mode for Hybrid Sales (H Mode)

In the H model, the manufacturer not only sets up a store on the e-commerce platform to directly sell products to consumers but also wholesales products to the e-commerce platform. The objective functions of the e-commerce platform, manufacturer, and government in the hybrid-mode ECLSC are, respectively,

(9) $$\prod_{E}^{H}=\rho P_{M}^{H}D_{M}^{H}+(P_{E}^{H}-w^{H})D_{E}^{H}+(B^{H}-A^{H})Q^{H}-S_{0}Q^{H}-SD_{M}^{H}$$

(10) $$\prod_{M}^{H}=(w^{H}-c_{n})D_{E}^{H}+(P_{M}^{H}-c_{n})D_{M}^{H}+(\Delta +g^{H}-B^{H}-c_{d})Q^{H}-\rho P_{M}^{H}D_{M}^{H}-h^{H}(D_{M}^{H}+D_{E}^{H})$$

(11) $$\prod_{G}^{H}=\prod_{M}^{H}+\prod_{E}^{H}+X^{H}+h^{H}(D_{M}^{H}+D_{E}^{H})-g^{H}Q^{H}-C(D_{M}^{H}+D_{E}^{H})+VQ^{H}$$

where $\Pi_{E}^{H}$ represents the profit of the e-commerce platform. The first item is the commission income from the manufacturer renting the platform, the second is the self-operated sales profit of the e-commerce platform, the third is the income from recycling WEEEs, the fourth is the cost paid by WEEEs, and the fifth is the operational cost of the e-commerce platform. $\Pi_{M}^{H}$ represents the manufacturer’s profit. The first item is the profit from wholesale products to the e-commerce platform, the second is the profit from product sales, the third is the income from recycling and processing WEEEs (equal to government subsidies minus the cost of recycling WEEEs from the e-commerce platform and then minus the cost of dismantling and processing WEEEs) and cost savings for remanufacturing, the fourth is the commission paid to the e-commerce platform, and the fifth is the processing fee levied by the government. $\Pi_{G}^{H}$ represents the government’s benefits (social welfare). The first item is the manufacturer’s profits, the second is the e-commerce platform’s profits, the third is consumer surplus, the fourth is the processing fee collected by the government, the fifth is the government’s subsidy for dismantling and processing WEEEs, the sixth is the environmental cost of consumer electrical and electronic products, and the seventh is the environmental benefit brought by recycling WEEEs.

Consumer surplus is the sum of consumer surplus in the forward supply chain and the reverse supply chain, namely,

(12) $$X_{}^{H}=\frac{\left(\alpha -\beta P_{M}^{H}-\beta_{1}(P_{M}^{H}-P_{E}^{H}))(\frac{\alpha }{\beta }-P_{M}^{H}\right)}{2}+\frac{\beta_{1}{\left(P_{M}^{H}-P_{E}^{H}\right)}^{2}}{2}+Q_{0}A^{H}+\frac{\delta {\left(A^{H}\right)}^{2}}{2}$$

According to the reverse-induction method, the optimal decision of the ECLSC members in the H model can be obtained:

$$g^{H^{*}}=\frac{(4V-3S_{0}-3c_{d}+3c_{n}-3c_{r})\delta +3Q_{0}}{\delta }$$

$$h^{H^{*}}=\frac{(8(C+c_{n}+S)+\beta_{1}S-4\alpha )(1-\rho )-4c_{n}\beta }{4\beta }$$

$$P_{E}^{H^{*}}=\frac{8(C+S+c_{n})\beta -2S\beta +\beta_{1}S}{8\beta }$$

$$A^{H^{*}}=V-S_{0}+c_{n}-c_{d}-c_{r}$$

$$w^{H^{*}}=\frac{8\beta (1-\rho )(C+c_{n}+S)+S(4\beta +\beta_{1}(1-\rho ))}{8\beta }$$

$$P_{M}^{H^{*}}=\frac{8(C+c_{n}+S)\beta +\beta_{1}S}{8\beta }$$

$$B^{H^{*}}=\frac{(2V-S_{0}+2c_{n}-2c_{d}-2c_{r})\delta +Q_{0}}{\delta }$$

At this point, the manufacturer’s optimal profit is

$$\begin{array}{l} \prod_{M}^{H^{*}}=\frac{1}{64\beta \delta }(128\beta {\left(c_{r}-V+S_{0}+c_{d}-c_{n}\right)}^{2}\delta^{2}+(64\beta^{2}(1-\rho ){\left(C+c_{n}+S\right)}^{2}\\ +(16(1-\rho )(\beta_{1}S^{2}+(C+c_{n})S\beta_{1}-8\alpha (C+c_{n}+S))+8\beta_{1}S^{2}+256Q_{0}(V-S_{0}\\ -c_{d}+c_{n}-c_{r}))\beta -{\left(\beta_{1}S-8\alpha \right)}^{2}(1-\rho ))\delta +128Q_{0}^{2}\beta ) \end{array}$$

The optimal profit of the e-commerce platform is

$$\begin{array}{l} \prod_{E}^{H^{*}}=\frac{1}{64\beta \delta }(64\beta {\left(c_{r}-V+S_{0}+c_{d}-c_{n}\right)}^{2}\delta^{2}-64\beta^{2}({\left(C+c_{n}+S\right)}^{2}\rho -S(C+c_{n}+S))\\ +(-16(\beta_{1}S-4\alpha )(C+c_{n}+S)\rho +12\beta_{1}S^{2}-64S\alpha +128Q_{0}(V-S_{0}-c_{d}+c_{n}-c_{r}))\beta \\ -\beta_{1}S\rho (\beta_{1}S-8\alpha ))\delta +64\beta Q_{0}^{2}) \end{array}$$

The government’s optimal social welfare is

$$\begin{array}{l} \prod_{G}^{H^{*}}=\frac{1}{128\beta }(64\beta^{2}{\left(C+c_{n}+S\right)}^{2}+(64\delta {\left(c_{r}-V+S_{0}+c_{d}-c_{n}\right)}^{2}+128(V-S_{0}-c_{d}+c_{n}\\ -c_{r})Q_{0}+44\beta_{1}S^{2}+(16(C+c_{n})\beta_{1}-128\alpha )S-128\alpha (C+c_{n}))\beta +{\left(\beta_{1}S-8\alpha \right)}^{2}) \end{array}$$

Consumer surplus is

$$\begin{array}{l} X^{H^{*}}=\frac{1}{128\beta }(64\beta^{2}{\left(C+c_{n}+S\right)}^{2}+(64\delta {\left(c_{r}-V+S_{0}+c_{d}-c_{n}\right)}^{2}+128(V-S_{0}-c_{d}+c_{n}\\ -c_{r})Q_{0}+36\beta_{1}S^{2}+(32(C+c_{n})\beta_{1}-128\alpha )S-128\alpha (C+c_{n}))\beta +{\left(2\beta_{1}S-8\alpha \right)}^{2}-S^{2}\beta_{1}^{2}) \end{array}$$

## 5. Comparative Analysis of the ECLSC’s Optimal Decisions under the Three Sales Cooperation Modes

**Proposition 1.**  *The government subsidies for dismantling WEEEs are equal under different cooperation modes, $g^{T^{*}}=g^{Z^{*}}=g^{H^{*}}$, and $g^{j}$ is not associated with C but has a positive correlation with V.*

All of the proofs are in “Appendix B”.

Proposition 1 shows that the subsidy standard given by the government for WEEE dismantling will not vary depending on different sales cooperation modes. In other words, as long as the same electrical and electronic products are disassembled, the subsidy standard should be the same under different modes. In addition, C is the environmental cost of consuming a unit of electrical and electronic products, and V is the environmental benefit of dismantling and processing a unit of WEEEs. This proposition means the unit subsidy standard given by the government for WEEE treatment does not need to consider the environmental cost generated by consumer unit products but only the environmental benefit brought by the dismantling treatment. In the same time, the higher the benefit, the higher the subsidy standard.

**Proposition 2.**  *When $C\leq \frac{\left(\left(-2S-2c_{n}\right)\rho +2S-2c_{n}\right)\beta +\alpha \left(\rho +2\right)}{2\beta \left(\rho +1\right)}$, there is a relation: $h^{H^{*}}>h^{T^{*}}\geq h^{Z^{*}}$. When $\frac{\left(\left(-2S-2c_{n}\right)\rho +2S-2c_{n}\right)\beta +\alpha \left(\rho +2\right)}{2\beta \left(\rho +1\right)}<C\leq \frac{\left(\left(-8S-8c_{n}\right)\rho +8S-8c_{n}\right)\beta +\left(-\beta_{1}S+4\alpha \right)\rho +\beta_{1}S+8\alpha }{8\beta \left(\rho +1\right)}$, there is a relation: $h^{H^{*}}\geq h^{Z^{*}}>h^{T^{*}}$. When $C>\frac{\left(\left(-8S-8c_{n}\right)\rho +8S-8c_{n}\right)\beta +\left(-\beta_{1}S+4\alpha \right)\rho +\beta_{1}S+8\alpha }{8\beta \left(\rho +1\right)}$, there is a relation: $h^{Z^{*}}>h^{H^{*}}>h^{T^{*}}$.*

Proposition 2 shows that the cooperation modes between manufacturers and e-commerce platforms affect the government’s levying of a fixed fee amount for electrical and electronic products. Under the hybrid sales cooperation mode (H mode), the fee should be higher than in the manufacturer’s direct sales cooperation mode (T mode). The relative size of the fee for WEEE disposal levied under different sales cooperation modes between manufacturers and e-commerce platforms is related to the environmental cost (C). When $C\leq \frac{\left(\left(-2S-2c_{n}\right)\rho +2S-2c_{n}\right)\beta +\alpha \left(\rho +2\right)}{2\beta \left(\rho +1\right)}$, electrical and electronic products are relatively environmentally friendly products. At this time, among the three sales cooperation modes, the fee for WEEE disposal levied under mode Z is the lowest. When $C>\frac{\left(\left(-8S-8c_{n}\right)\rho +8S-8c_{n}\right)\beta +\left(-\beta_{1}S+4\alpha \right)\rho +\beta_{1}S+8\alpha }{8\beta \left(\rho +1\right)}$, products have a great effect on environmental protection. At this time, among the three modes of sales cooperation, the fee for WEEE disposal levied under model Z is the highest.

**Proposition 3.**  *When $0<C\leq \frac{\left(\left(-8S-8c_{n}\right)\rho +12S-8c_{n}\right)\beta -{S\rho \beta }_{1}+\beta_{1}S+8\alpha }{8\beta \left(\rho +1\right)}$, $w^{H^{*}}\geq w^{Z^{*}}$; when $C>\frac{\left(\left(-8S-8c_{n}\right)\rho +12S-8c_{n}\right)\beta -{S\rho \beta }_{1}+\beta_{1}S+8\alpha }{8\beta \left(\rho +1\right)}$, $w^{H^{*}}<w^{Z^{*}}$.*

Proposition 3 shows that the manufacturer’s wholesale prices should be different under different sales cooperation modes, and the relative size is related to environmental costs. When environmental cost C is low, the manufacturer’s wholesale price in the H model is higher than in the Z model; when environmental cost is high, the reverse is true.

**Proposition 4.**  *The transfer payment price by manufacturer to e-commerce platform for recycling WEEEs and the price paid by e-commerce platform to consumer for recycling WEEEs are equal under different modes, i.e., $B^{T^{*}}=B^{Z^{*}}=B^{H^{*}}$, $A^{T^{*}}=A^{Z^{*}}=A^{H^{*}}$; both types of prices are positively related to environmental benefits.*

Proposition 4 shows that in the ECLSC of electrical and electronic products, different sales cooperation modes between manufacturers and e-commerce platforms will not affect the recycling price of WEEEs. Under the three different sales cooperation modes, the price of WEEEs recovered from consumers by e-commerce platforms is the same while the transfer payment of WEEEs obtained from e-commerce platforms by manufacturers is also the same.

**Proposition 5.**  *The sales price of the electronic and electric products under different modes has the relationship $P_{M}^{H}>P_{E}^{H}>P_{M}^{T}>P_{E}^{Z}$; sales price is positively related to environmental cost.*

Proposition 5 indicates that when the sales cooperation modes between manufacturers and e-commerce platforms are different, the sales prices of electrical and electronic products are different. First, for manufacturers, the sales price of electrical and electronic products under the H mode should be higher than that under the T mode. Second, for e-commerce platforms, the sales price under the H mode is higher than that under the Z mode. Third, to ensure the brand effect, the manufacturer’s direct sales price is higher than the platform’s self-operated sales price under the H mode.

For the ECLSC, Proposition 5 also shows that the sales price of electrical and electronic products is the highest under the H model and the lowest under the Z mode. Obviously, the Z mode will bring greater benefits to consumers when after-sales product service is guaranteed.

**Proposition 6.**  *Under different sales cooperation modes between manufacturers and e-commerce platforms, the government’s social welfare has the following relationship:*

(1) *When $0<C\leq \frac{\left(-44S-16c_{n}\right)\beta -\beta_{1}S+16\alpha }{16\beta }$, $\prod_{G}^{H^{*}}\leq \prod_{G}^{T^{*}}<\prod_{G}^{Z^{*}}$.*
(2) *When $\frac{\left(-44S-16c_{n}\right)\beta -\beta_{1}S+16\alpha }{16\beta }<C\leq \frac{\left(-64S-128c_{n}\right)\beta^{2}+\left(\left(-44S-16c_{n}\right)\beta_{1}+128\alpha \right)\beta -{S\beta }_{1}^{2}+{16\alpha \beta }_{1}}{{128\beta }^{2}+{16\beta }_{1}\beta }$, $\prod_{G}^{T^{*}}<\prod_{G}^{H^{*}}\leq \prod_{G}^{Z^{*}}$.*
(3) *When $\frac{\left(-64S-128c_{n}\right)\beta^{2}+\left(\left(-44S-16c_{n}\right)\beta_{1}+128\alpha \right)\beta -{S\beta }_{1}^{2}+{16\alpha \beta }_{1}}{{128\beta }^{2}+{16\beta }_{1}\beta }<C\leq \frac{\left(-S-2c_{n}\right)\beta +2\alpha }{2\beta }$, $\prod_{G}^{T^{*}}\leq \prod_{G}^{Z^{*}}<\prod_{G}^{H^{*}}$.*
(4) *When $C>\frac{\left(-S-2c_{n}\right)\beta +2\alpha }{2\beta }$, $\prod_{G}^{Z^{*}}<\prod_{G}^{T^{*}}<\prod_{G}^{H^{*}}$.*

Proposition 6 shows that different sales cooperation modes between manufacturers and e-commerce platforms lead to different social welfare, and social welfare is related to environmental costs. For environmentally friendly electrical and electronic products, the government can obtain the largest social welfare under the Z mode, and it can get the minimum social welfare under the H model. For electrical and electronic products with moderately environmentally friendly properties, the government gets the least social welfare under the T mode, and social welfare in the other two models is higher than T mode. For electrical and electronic products causing serious environmental pollution, the government obtains the largest social welfare under the H mode while the Z mode has the smallest social welfare, which is the opposite of the situation involving environmentally friendly electrical and electronic products.

**Proposition 7.**  *Under different sales cooperation modes between manufacturers and e-commerce platforms, consumer surplus has the following relationship:*

(1) *When $0<C\leq \frac{\left(-36S-32c_{n}\right)\beta -{3\beta }_{1}S+32\alpha }{32\beta }$, $X^{H^{*}}\leq X^{T^{*}}<X^{Z^{*}}$.*
(2) *When $\frac{\left(-36S-32c_{n}\right)\beta -{3\beta }_{1}S+32\alpha }{32\beta }<C\leq \frac{\left(-64S-128c_{n}\right)\beta^{2}+\left(\left(-36S-32c_{n}\right)\beta_{1}+128\alpha \right)\beta -{3S\beta }_{1}^{2}+{32\alpha \beta }_{1}}{{128\beta }^{2}+{32\beta }_{1}\beta }$, $X^{T^{*}}\leq X^{H^{*}}<X^{Z^{*}}$.*
(3)
$\frac{\left(-64S-128c_{n}\right)\beta^{2}+\left(\left(-36S-32c_{n}\right)\beta_{1}+128\alpha \right)\beta -{3S\beta }_{1}^{2}+{32\alpha \beta }_{1}}{{128\beta }^{2}+{32\beta }_{1}\beta }<C\leq \frac{\left(-S-2c_{n}\right)\beta +2\alpha }{2\beta }$ *,* $X^{T^{*}}\leq X^{Z^{*}}<X^{H^{*}}$ *.*
(4)
$C>\frac{\left(-S-2c_{n}\right)\beta +2\alpha }{2\beta }$ *,* $X^{Z^{*}}\leq X^{T^{*}}<X^{H^{*}}$ *.*

Proposition 7 shows that the sales cooperation mode between manufacturers and e-commerce platforms is different, consumer surplus is also different, and consumer surplus is related to environmental costs. For environmentally friendly electrical and electronic products, the Z mode has the largest consumer surplus, while the H mode has the smallest. For electrical and electronic products with moderately environmentally friendly properties, the T mode has the minimum consumer surplus and is lower than the other two modes. For electrical and electronic products causing serious environmental pollution, consumer surplus is larger under the H mode while this Z mode has the smallest consumer surplus. The opposite is the case for environmentally friendly electrical and electronic products. These conclusions are similar to those of proposition 6, except that the scope of environmental cost C of electrical and electronic products is different for the consumer unit that defines the environmental friendliness of electrical and electronic products.

**Proposition 8.**  *Under different sales cooperation modes between manufacturers and e-commerce platforms, manufacturers’ profits, e-commerce platform profits, social welfare, and consumer surplus all increase with the increase in environmental benefit V.*

Proposition 8 shows that regardless of the sales cooperation mode, the greater the environmental benefit of recycling and processing WEEEs, the greater the manufacturer’s profit, e-commerce platform’s profit, social welfare, and consumer surplus. This shows that improving recycling technology and increasing the environmental benefits of recycling WEEEs will increase the benefits of all members of the supply chain.

## 6. Numerical Simulation

MATLAB was used to analyze the change in the environmental cost C of consumer electrical and electronic products under different sales cooperation modes for processing fees h (levied by the government), recycling WEEE subsidies g, wholesale price w, sales price P, social welfare $\Pi_{G}$, manufacturer’s profit $\Pi_{M}$, network platform margin $\Pi_{E}$, and change in consumer surplus X.

Taking one electrical and electronic product as an example, the parameter values of the model are assumed as follows. Based on the empirical sales data of a local brand smartphone, we assume α=800,000, $\beta_{1}=10,000$, $c_{n}=700$, $c_{r}=550$, and $c_{d}=10$. In addition, based on the practices observed in JD.com, a giant e-commerce platform in China, we assume ρ=0.07, and we further assume $S_{0}=20$, S=10, $Q_{0}=10,000$, δ=100, and β=1000.

### 6.1. The Impact of Unit Environmental Cost

**Figure 4:**
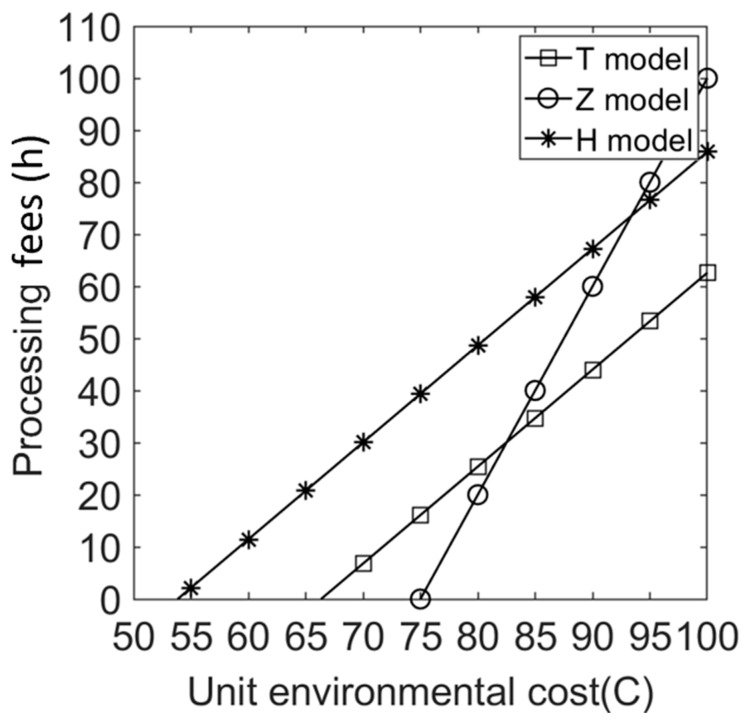
Effects of environmental costs on product processing fees under different cooperation modes.

**Figure 5:**
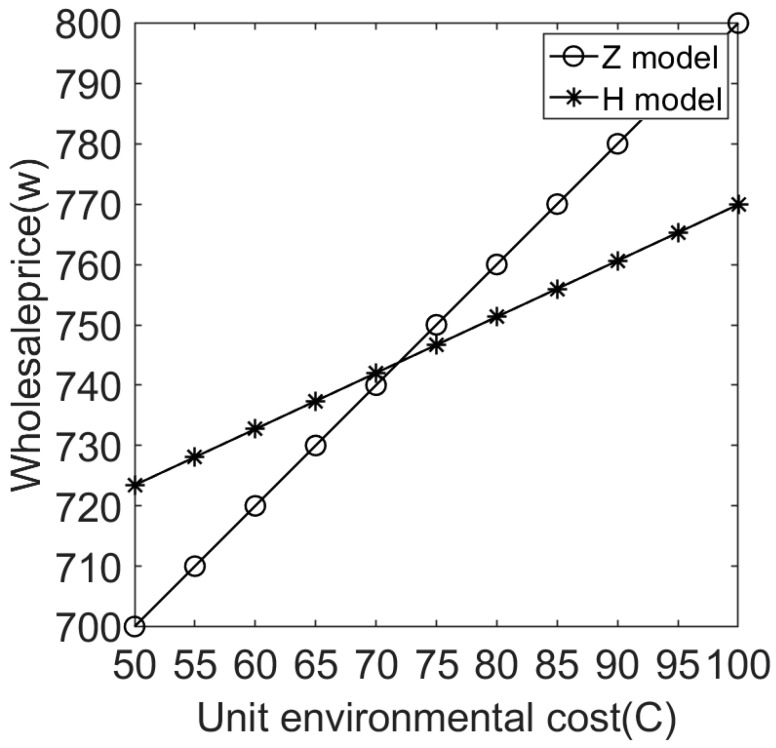
Effects of environmental costs on wholesale prices under different sales cooperation modes.

**Figure 6:**
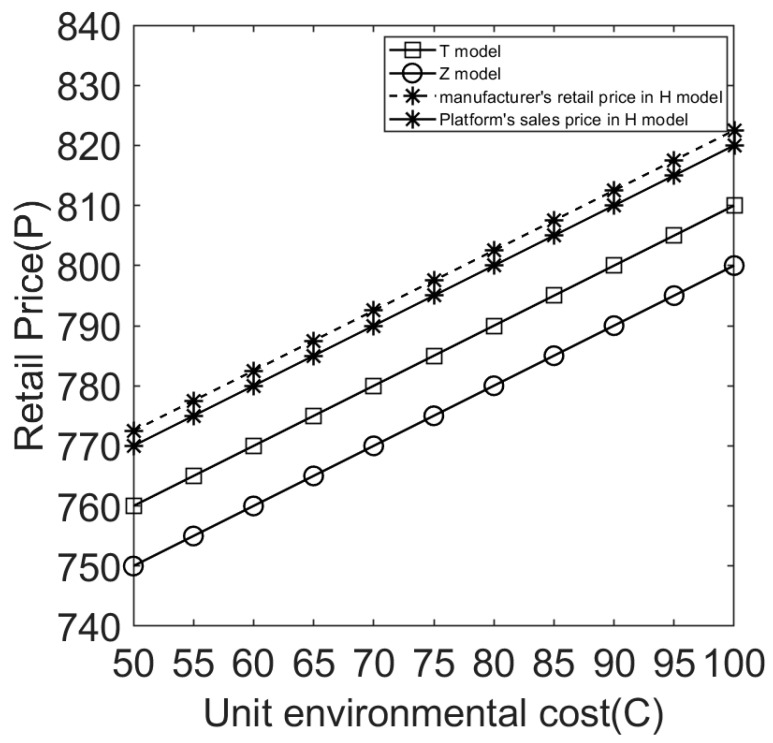
Effect of environmental costs on retail prices under different sales cooperation modes.

**Figure 7:**
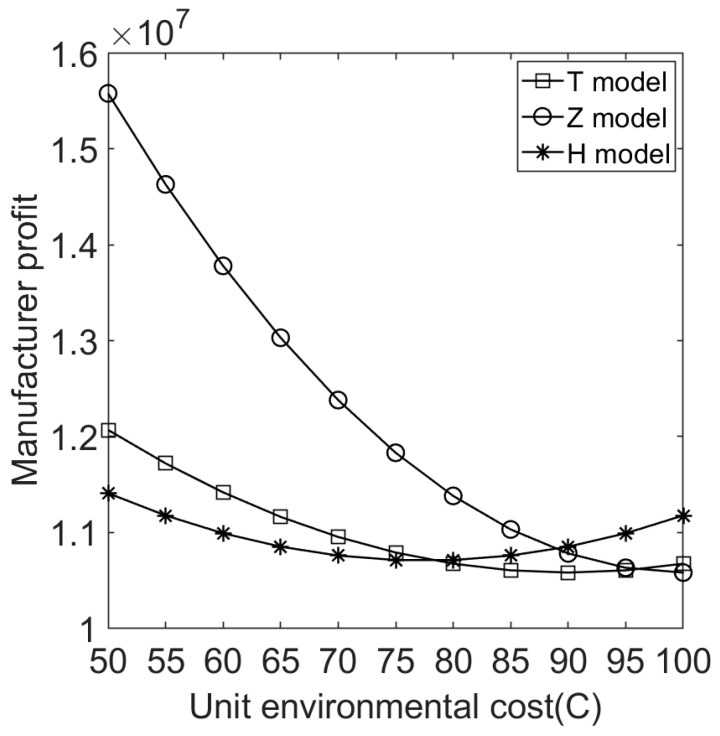
Effects of environmental costs on the manufacturer’s profits under different sales cooperation modes.

**Figure 8:**
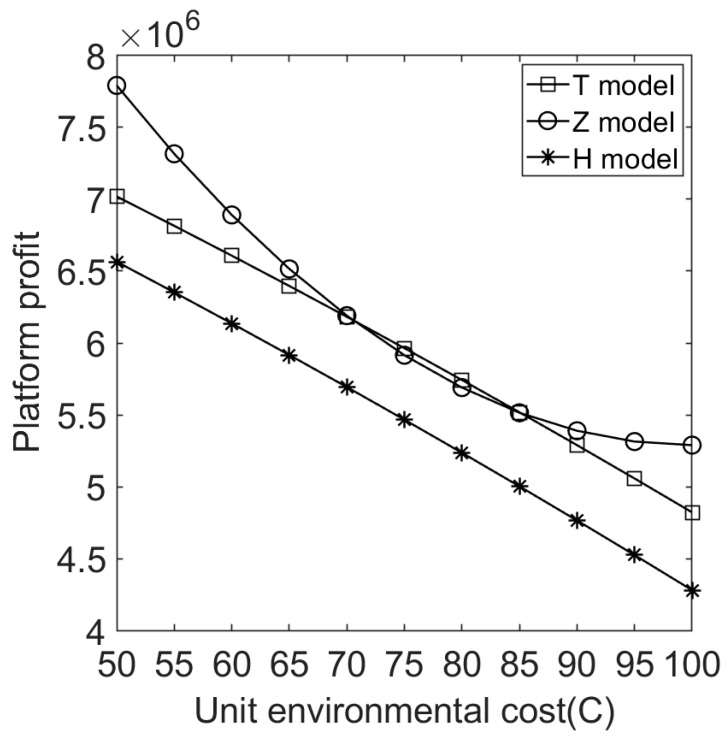
Effect of environmental costs on e-commerce platform profits under different sales cooperation modes.

**Figure 9:**
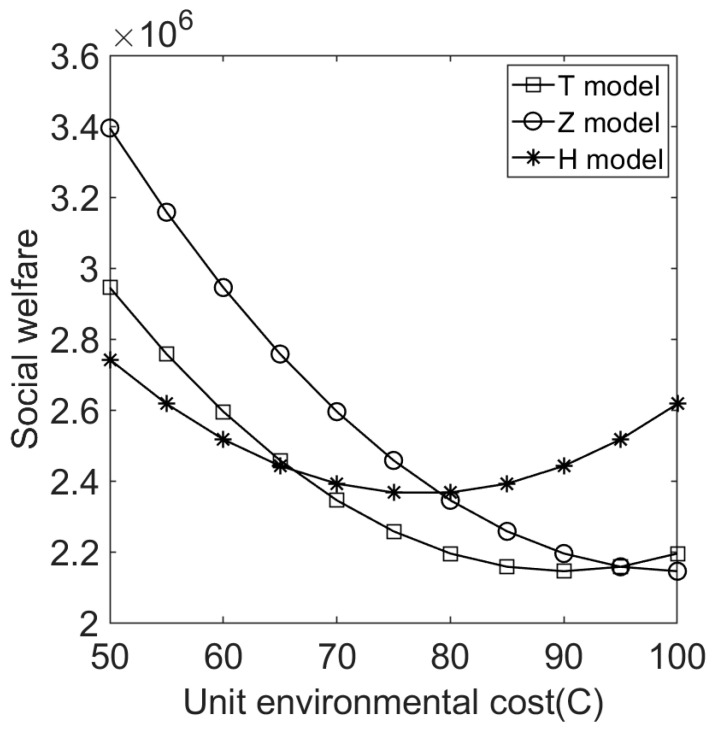
Effect of environmental cost on social welfare under different sales cooperation modes.

We let V=10 and C∈[50,100]. Figure 4, Figure 5, Figure 6, Figure 7, Figure 8 and Figure 9 show the impact of the unit environmental on processing fees, wholesale prices, retail prices, and profits.

(1) Effects of environmental costs on product processing fees under different sales cooperation modes.

Figure 4 shows that, under different sales cooperation modes, the minimum environmental costs required by the government for levying processing fees vary widely. That is, only when the environmental costs of consumer products reach a certain threshold will the government begin to levy processing fees on electrical and electronic products. This means two things. First, the environmental cost caused by the consumption of each kind of electrical and electronic product is basically the same. Under model H, costs levied by the government for processing fees are minimal; under model T, they are higher. Under model Z, the costs levied by the government for processing fees are the highest. To avoid paying product processing fees, manufacturers should evaluate the environmental costs of their products when cooperating with the platform and choose models T and Z instead of model H. Second, the manufacturer should produce energy-saving, environmentally friendly products to reduce the environmental costs of product consumption and obtain a disposal-fee exemption.

Figure 4 also shows that the amount of processing fees levied by the government increases with increases in environmental cost C under all models. The amount of levied processing fees under model H is always greater than that under model T; under model Z, it varies widely with the environmental cost of the product. When environmental costs reach a certain threshold, the amount of levied processing fees under model H is the largest. As environmental costs continue to increase, the amount of levied processing fees is higher than in the other two models. Proposition 2 is consistent with this conclusion.

(2) Effects of environmental costs on wholesale prices under different sales cooperation modes.

Figure 5 shows that the wholesale price of electrical and electronic products increases with the increase in environmental costs caused by consumption. This means the higher the environmental cost, the higher the product wholesale price. This is because manufacturers must pay more processing fees as environmental costs increase; thus, some losses can be compensated for by raising wholesale prices.

It can also be seen in Figure 5 that when environmental costs are lower (i.e., environmentally friendly products), the wholesale price of model H is greater than that of model Z. However, when environmental costs exceed a certain threshold (i.e., products with greater bad environmental effects), the wholesale price of model H is smaller than that of model Z. This is consistent Proposition 3.

(3) Effect of environmental costs on retail prices under different sales cooperation modes.

Figure 6 shows a positive correlation between the selling price of electrical and electronic products and the environmental costs of products. The higher the environmental cost of a product, the higher the retail price. This is because when a product’s environmental costs are higher, the manufacturer will pay more processing fees; thus, retail prices will also be higher to ensure profits. Figure 6 also clearly shows that the retail price of electrical and electronic products under model H is higher than that under models T and Z. Under model H, the manufacturer’s retail price is higher than that of the e-commerce platform. The manufacturer’s retail price under model T is higher than that of the e-commerce platform under model Z. These conclusions are consistent with Proposition 5.

(4) Effect of environmental costs on the profits of manufacturers and e-commerce platforms under different sales cooperation modes.

Figure 7 shows that a product’s environmental cost and different sales cooperation modes greatly affect the manufacturer’s profits. When manufacturers produce and sell products with different environmental costs, their profits do not always decrease with increased environmental costs. When the environmental costs are between different intervals, the manufacturer’s profit is different under different sales cooperation modes. For environmentally friendly products (i.e., the product’s environmental cost is relatively small), the manufacturer adopts model Z to get the most profit and model H to get the least profit (thus, multichannel sales are not profitable for the manufacturers, which is interesting). This is related to the fact that under model Z, the government does not levy product processing fees on products with low environmental costs.

Figure 8 shows that a product’s environmental costs and the e-commerce platform’s profit are affected by the sales cooperation mode with the manufacturer. The e-commerce platform’s profits are higher under model T than under model H (multiple channels diversify profit sources). With increased environmental costs, the e-commerce platform’s profits will be reduced under models T and H. This means that products with high environmental pollution will reduce the e-commerce platform’s profits.

(5) Effect of environmental cost on social welfare and consumer surplus under different sales cooperation modes.

Figure 9 shows that given the environmental costs of products, different sales cooperation modes will affect social welfare differently. The relative amount of government social welfare is different under the three modes with different environmental costs of products. For environmentally friendly products (environmental cost is low), model Z can bring higher social welfare, while model H can bring more social welfare when environmental pollution is relatively large (environmental cost is relatively high). Proposition 6 is consistent with this conclusion. This is related to the government’s policies of processing fees, remanufacturing subsidies, and pricing strategies when there are environmental costs.

**Figure 10:**
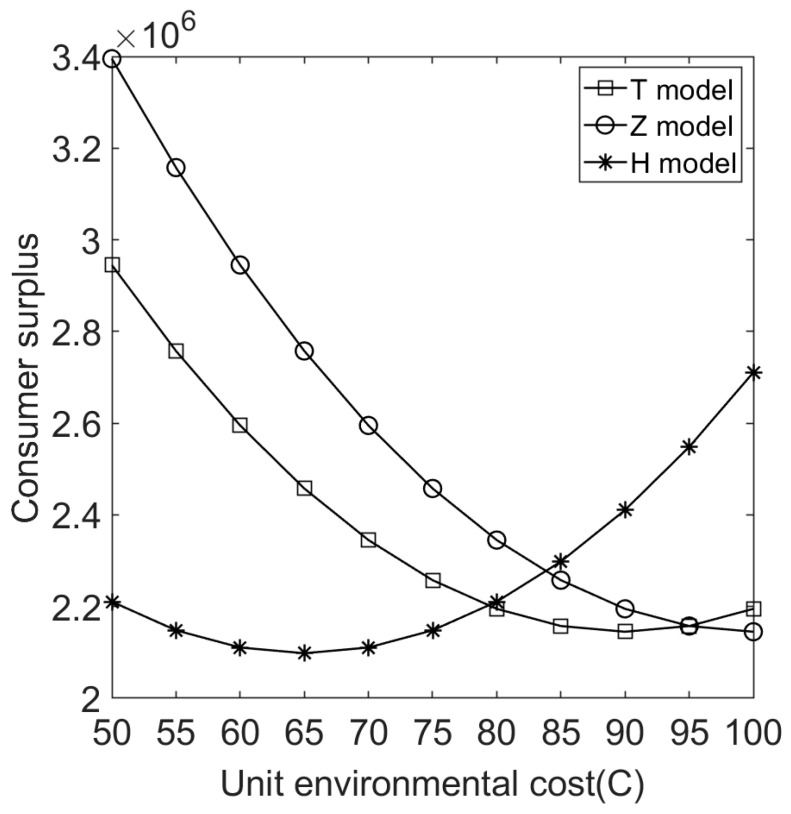
Effect of environmental cost on consumer surplus under different sales cooperation modes.

Figure 10 shows that the effect of environmental costs on consumer surplus under different sales cooperation modes is similar to the relationship and analysis shown in Figure 9.

### 6.2. The Impact of Unit Environmental Benefit

**Figure 11:**
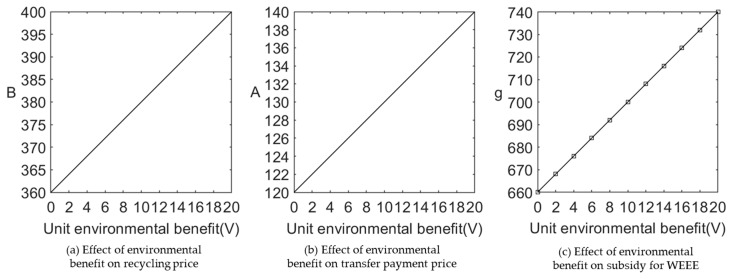
Effect of environmental benefit on recycling price, transfer payment price, and subsidy under different sales cooperation modes.

**Figure 12:**
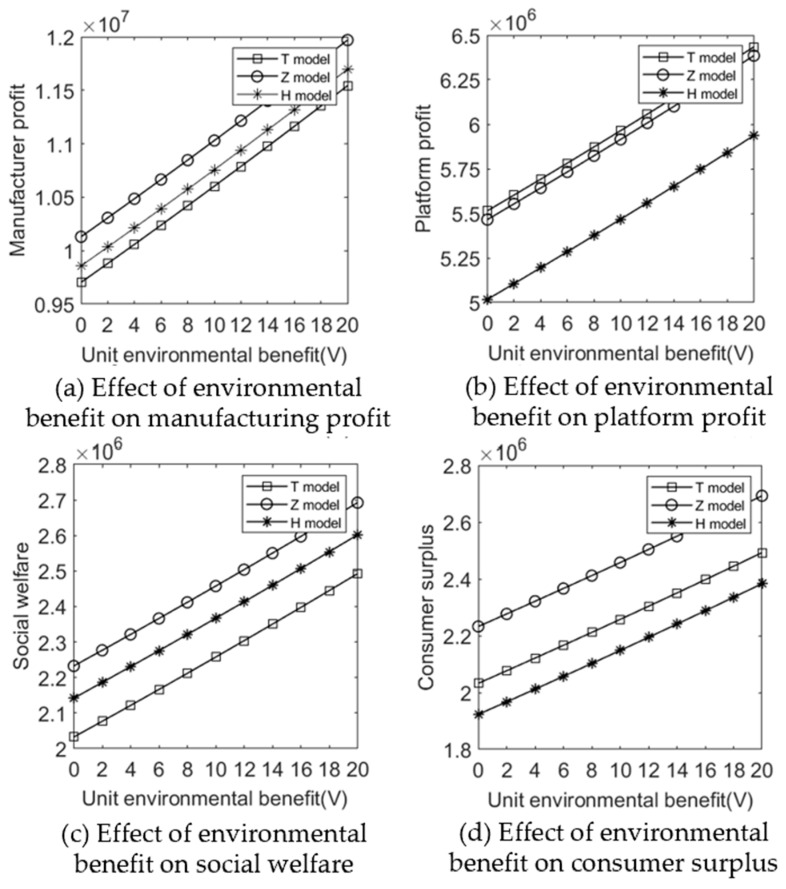
Effect of environmental benefit on profits and social welfare under different sales cooperation modes.

We let V∈[0,20] and C=75. Figure 11 and Figure 12 show the impact of the unit environmental benefit on recycling price paid by e-commerce platform for WEEE, wholesale prices, retail prices, and profits.

(1) Effect of environmental benefit on recycling price, transfer payment price, and subsidy under different sales cooperation modes.

Figure 11 shows that environmental benefits are positively related to the WEEE recycling prices, transfer payment prices, and government subsidies. This is mainly because the higher the environmental benefits, the more valuable the WEEE recycling and processing activities are. Therefore, the government will increase subsidies for this activity, manufacturers are also willing to pay higher transfer payment prices to purchase WEEE from the platform, and the platform will choose to pass more benefits to consumers.

(2) Effect of environmental benefit on profits and social welfare under different sales cooperation modes.

Figure 12 shows that as the environmental benefits of recycling and processing waste electrical and electronic equipment increase, the profits of manufacturers in all three modes also increase. Combined with Figure 10, it can be seen that although the government provides higher subsidies, this does not lead to a decrease in social welfare but rather to an increase as environmental benefits increase. Manufacturers and platforms also enjoy the dividends of increased environmental benefits. Therefore, the application of new processing technologies and standardized processes to increase environmental benefits is very valuable to society, and the application and development of recycling technologies should be encouraged.

## 7. Result Discussion

Based on the analysis above, the result discussions are detailed below.

First, subsidy standards for WEEE dismantling should not be affected by changes in environmental costs and sales cooperation modes. Rather, higher subsidies should be given for WEEE dismantling if recycling brings greater environmental benefits. Meanwhile, the government should not levy processing fees on products under all conditions; rather, a limit should be placed on the amount of environmental damage (cost) caused by product consumption (if the limit is exceeded, a processing fee will be collected). As a condition for the introduction of a fee for product processing, sales under different cooperation models should set different environmental damage (cost) limits. Model H introduces the minimum environmental cost limit required by the processing fees while model Z requires the highest environmental cost to levy processing fees. The amount of levied processing fees is also affected by environmental costs and sales cooperation modes. Enterprises can reasonably avoid processing fees by producing energy-saving, environmentally friendly products and choosing the appropriate sales cooperation mode. The amount of processing fees is positively related with the change in environmental costs. The greater the environmental loss (cost) caused by product consumption, the higher the processing fee. The environmental benefits of recycling should not be considered in levying processing fees for electrical and electronic products. The finding that government policy for processing fees should consider environmental influence of products is consistent with other research where the environmental effects (e.g., carbon emissions) of the products can influence the government tax or reward/penalty regulations [35,36], and we extend the previous publications by considering different cooperation modes.

Second, considering the government’s WEEE regulations, the wholesale and retail prices of electrical and electronic products will be affected by sales cooperation mode and environmental cost but not relevant to environmental benefit. The relative amount of wholesale prices under different sales cooperation modes is also related to environmental costs. When the threshold of the environmental cost is exceeded, the relationship on the relative amount will be reversed. The change in environmental cost will not affect the relative relation of product retail prices under different sales cooperation modes. Under model H, the retail prices of products are the highest (the manufacturer’s retail price is higher than that of the e-commerce platform), while under model Z, the retail price is the lowest, which will bring more benefits to consumers. As for the WEEE recovery price, the recovery price of the e-commerce platform and the transfer payment price of the manufacturer are not affected by the sales model and have nothing to do with environmental cost but are positively correlated with environmental benefit. In other words, the higher the environmental benefit brought from WEEE recycling, the higher the recovery price. Compared with previous studies, our result can validate their findings as we show that the sales cooperation mode can determine the pricing decisions of forward and reverse supply chain, which has been witnessed in previous literature [37]

Third, environmental costs under different sales cooperation modes will affect the profits of supply chain members, but increased environmental cost does not always decrease profits. Under different sales cooperation modes, the relative amount of corporate profit and social welfare is related to the range of environmental costs. For environmentally friendly products, multichannel sales do not necessarily increase a manufacturer’s profits. In addition, the greater the environmental benefit brought by WEEE recycling, the higher the profits of manufacturers and e-commerce platforms, and social welfare and consumer surplus will increase as well. From the perspective of government regulation and enterprise operations, it is a better choice to give priority to recycling WEEEs with large environmental benefits. The phenomenon that products’ environmental effect can influence different supply chain members, customers, or society is also witnessed in different studies [38,39]. Compared with them, our model extends the previous literature by considering both environmental cost and benefit on all relevant players together in an ECLSC cooperation angle. 

## 8. Conclusions

Currently, it is common that an e-commerce platform participates in the sale and recycling of electrical and electronic products. This study focused on three modes of cooperative sales between manufacturers and e-commerce platforms, considering governmental WEEE regulations and the environmental effects of products. A Stackelberg game model for ECLSC was constructed. Reverse induction was used to obtain the optimal decisions and objective functions of the manufacturer, e-commerce platform, and government. The different sales cooperation modes were compared for optimal decisions, profits, social welfare, and consumer surplus. Furthermore, the environmental effects of electrical and electronic products under the three sales cooperation modes were discussed in terms of decision-making, profits, and social welfare. Finally, simulation analysis was used to verify the results. We believe this study can contribute to the current literature by building stylized models to examine the relationship and decision-making among governments, e-commerce platforms, and manufacturers. In addition, this study has significant practical implications, as the findings of this study can facilitate the development of government policies, company pricing decisions, and recycling rules. 

This research is based on the assumption of complete information. The demand function and recovery function are deterministic situations that do not consider the sales of electrical and electronic products by manufacturers in traditional channels. Future research can be extended to include information asymmetry, uncertain demand functions and recovery amounts, and increased sales through traditional channels.

## Figures and Tables

**Figure A1:**
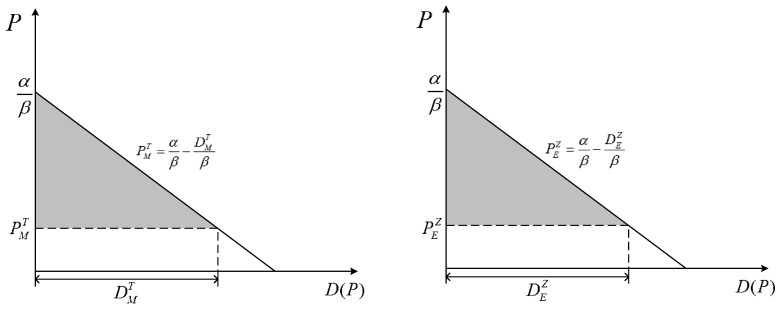
The consumer surplus under T mode (**left**) and Z mode (**right**) in the forward supply chain.

**Figure A2:**
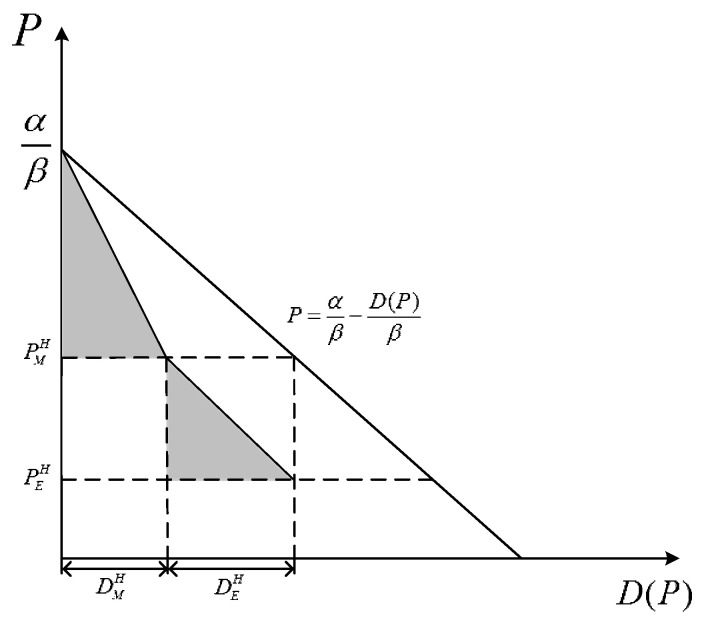
The consumer surplus under hybrid mode (H mode) in the forward supply chain.

**Figure A3:**
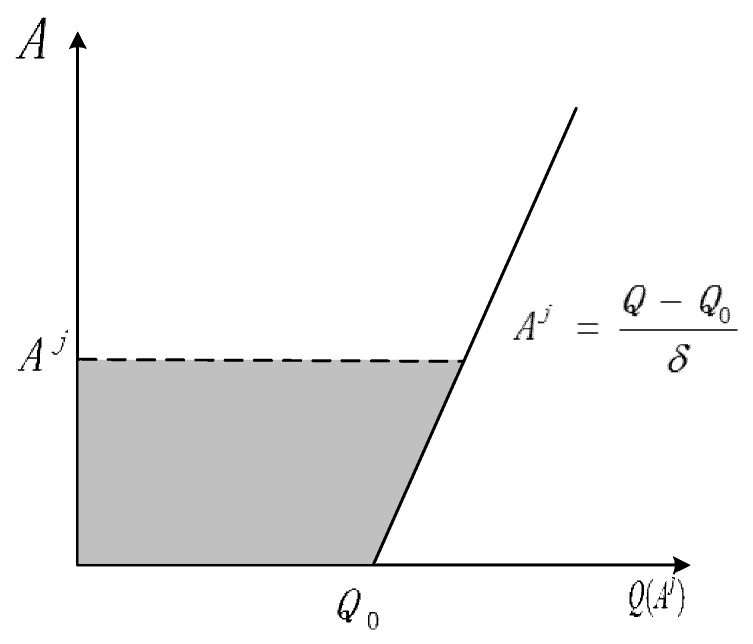
The consumer surplus in the reverse supply chain under three different modes.

## Data Availability

Not applicable.

## Nomenclature

**Parameter**	**Meaning**
D	Demand function of electrical and electronic products
α	Market capacity
β	Sensitivity coefficient of consumers to selling price
$\beta_{1}$	Sensitivity coefficient of consumers to the mean price between the manufacturer’s direct channel and self-operated platform channel
Q	WEEE recovery
$Q_{0}$	WEEE recovery of voluntary consumer recycling
δ	Sensitivity coefficient of consumers to recycling price
$c_{r}$	Unit production cost of remanufactured products
$c_{n}$	Unit production cost of new products
Δ	$\Delta =c_{n}-c_{r}$ cost saving of remanufacturing, Δ>0
ρ	Commission rate paid by manufacturer to e-commerce platform (0<ρ<1) as an exogenous variable
$c_{d}$	Manufacturer’s unit cost for WEEE dismantling
S	E-commerce platform provides manufacturers with the unit cost of the sales platform, $0<S<\rho P_{M}^{j}$
$S_{0}$	Unit cost of WEEE recovery by e-commerce platform
C	Environmental cost of consuming each electrical and electronic product
V	Environmental benefit of recycling each WEEE
Π	Profit function of supply chain members or utility function of government (social welfare)
X	Consumer surplus
${\left(\;\right)}_{i}^{j}$	i∈{M,E,G} represents supply chain members, where M represents manufacturer, E represents e-commerce platform, and G represents government j∈{T,Z,H} represents sales cooperation modes, where T represents direct sales mode, Z represents wholesale mode, and H represents hybrid mode.
**Decision** **Variable**	**Meaning**
w	Wholesale price of electrical and electronic products
P	Sales price of electrical and electronic products
A	Price paid by e-commerce platform to consumers for recycling WEEEs
B	Transfer payment price by manufacturer to e-commerce platform for recycling WEEEs
g	Government subsidies for dismantling WEEEs
h	Processing fee levied by the government on the production of electrical and electronic products per unit

## Appendix A. Calculation of Consumer Surplus

In the forward supply chain, under the direct sales and wholesale sales ECLSC models, the demand for electrical and electronic products is, respectively, $D_{M}^{T}=\alpha -\beta P_{M}^{T},\;D_{E}^{Z}=\alpha -\beta P_{E}^{Z}$, which is a function of prices $P_{M}^{T}$ and $P_{E}^{Z}$. Therefore, consumers can accept the upper limit of price $\frac{\alpha }{\beta }$. Under these two models, consumer surplus in the forward supply chain is shown in the shadowed areas in the Figure A1 below.

Therefore, the consumer surplus of the forward supply chain is as follows:

$$\frac{1}{2}D_{M}^{T}(\frac{\alpha }{\beta }-P_{M}^{T})=\frac{\alpha^{2}}{2\beta }-\alpha P_{M}^{T}+\frac{\beta {\left(P_{M}^{T}\right)}^{2}}{2}$$

$$\frac{1}{2}D_{E}^{Z}(\frac{\alpha }{\beta }-P_{E}^{Z})=\frac{\alpha^{2}}{2\beta }-\alpha P_{E}^{Z}+\frac{\beta {\left(P_{E}^{Z}\right)}^{2}}{2}$$

In the hybrid sales ECLSC model, the direct channel and self-operated channel are, respectively, $D_{M}^{H}=\alpha -\beta P_{M}^{H}-\beta_{1}\left(P_{M}^{H}-P_{E}^{H}\right)$ and $D_{E}^{H}=\beta_{1}\left(P_{M}^{H}-P_{E}^{H}\right)$, which is a function of prices $P_{M}^{H}$ and $P_{E}^{H}$. The total demand is $D_{M}^{H}+D_{E}^{H}=\alpha -\beta P_{M}^{H}$. Therefore, the upper limit of the price consumers can accept for purchasing electrical and electronic products through direct channels is $\frac{\alpha }{\beta }$. Because $P_{M}^{H}\geq P_{E}^{H}$, the upper price limit consumers can accept for purchasing electrical and electronic products through self-run channels is $P_{M}^{H}$. For the forward supply chain, the consumer surplus of self-operated channels and direct channels are shown in the shadowed areas in the Figure A2 below.

In the hybrid ECLSC model, the forward supply chain consumer surplus is

$$\begin{array}{l} \frac{1}{2}D_{M}^{H}(\frac{\alpha }{\beta }-P_{M}^{H})+\frac{1}{2}D_{E}^{H}(P_{M}^{H}-P_{E}^{H})\\ =\frac{1}{2}D_{M}^{H}(\frac{\alpha }{\beta }-P_{d})+\frac{{\left(D_{E}^{H}\right)}^{2}}{2\beta_{1}}\\ =\frac{\left(\alpha -\beta P_{M}^{H}-\beta_{1}(P_{M}^{H}-P_{E}^{H}))(\frac{\alpha }{\beta }-P_{M}^{H}\right)}{2}+\frac{\beta_{1}{\left(P_{M}^{H}-P_{E}^{H}\right)}^{2}}{2} \end{array}$$

In the reverse supply chain, the number of WEEEs recovered by the e-commerce platform is $Q^{j}=Q_{0}+\delta A^{j}$, which is a function of the recovery price. When the e-commerce platform recovers WEEEs, the minimum recovery price acceptable to consumers is 0. The consumer surplus in the reverse supply chain is shown in the shadowed area in the Figure A3 below.

That is, the consumer surplus of the reverse supply chain is

$$\begin{array}{l} \frac{1}{2}(Q_{0}+Q_{0}+\delta A^{j})A^{j}\\ =Q_{0}A^{j}+\frac{\delta {\left(A^{j}\right)}^{2}}{2} \end{array}$$

In summary, in the case of the direct-sales ECLSC model and the self-operating ECLSC model, the consumer surplus is, respectively,

$$X_{}^{T}=\frac{\alpha^{2}}{2\beta }-\alpha P_{M}^{T}+\frac{\beta {\left(P_{M}^{T}\right)}^{2}}{2}+Q_{0}A^{T}+\frac{\delta {\left(A^{T}\right)}^{2}}{2}$$

$$X^{Z}=\frac{\alpha^{2}}{2\beta }-\alpha P_{E}^{Z}+\frac{\beta {\left(P_{E}^{Z}\right)}^{2}}{2}+Q_{0}A^{Z}+\frac{\delta {\left(A^{Z}\right)}^{2}}{2}$$

In the hybrid ECLSC model, the consumer surplus is

$$X_{}^{H}=\frac{\left(\alpha -\beta P_{M}^{H}-\beta_{1}(P_{M}^{H}-P_{E}^{H}))(\frac{\alpha }{\beta }-P_{M}^{H}\right)}{2}+\frac{\beta_{1}{\left(P_{M}^{H}-P_{E}^{H}\right)}^{2}}{2}+Q_{0}A^{H}+\frac{\delta {\left(A^{H}\right)}^{2}}{2}$$

## Appendix B. Proofs

**Proof of Proposition 1.** $\frac{\partial g^{T^{*}}}{\partial V}=\frac{\partial g^{Z^{*}}}{\partial V}=\frac{\partial g^{H^{*}}}{\partial V}=4>0$; proposition 1 can thus be proven.□

**Proof of Proposition 2.** $h^{H^{*}}-h^{T^{*}}=\frac{\beta_{1}S(1-\rho )}{4\beta }$, when 0<ρ<1, $h^{H^{*}}>h^{T^{*}}$. $h^{H^{*}}-h^{Z^{*}}=\frac{8((1-\rho )(C+S+c_{n})-2(C+c_{n}))\beta +\beta_{1}S(1-\rho )+4\alpha (2+\rho )}{4\beta }$, if $C\leq \frac{\left(\left(-8S-8c_{n}\right)\rho +8S-8c_{n}\right)\beta +\left(-\beta_{1}S+4\alpha \right)\rho +\beta_{1}S+8\alpha }{8\beta \left(\rho +1\right)}$, $h^{H^{*}}\geq h^{Z^{*}}$; otherwise, $h^{H^{*}}<h^{Z^{*}}$. $h^{T^{*}}-h^{Z^{*}}=\frac{2((1-\rho )(C+S+c_{n})-2(C+c_{n}))\beta +2\alpha (2+\rho )}{\beta }$. If $C\leq \frac{\left(\left(-2S-2c_{n}\right)\rho +2S-2c_{n}\right)\beta +\alpha \left(\rho +2\right)}{2\beta \left(\rho +1\right)}$, $h^{T^{*}}\geq h^{Z^{*}}$; otherwise, $h^{T^{*}}<h^{Z^{*}}$.

$\frac{\left(\left(-8S-8c_{n}\right)\rho +8S-8c_{n}\right)\beta +\left(-\beta_{1}S+4\alpha \right)\rho +\beta_{1}S+8\alpha }{8\beta \left(\rho +1\right)}-\frac{\left(\left(-2S-2c_{n}\right)\rho +2S-2c_{n}\right)\beta +\alpha \left(\rho +2\right)}{2\beta \left(\rho +1\right)}=-\frac{S\left(\rho -1\right)\beta_{1}}{8\beta \left(\rho +1\right)}>0$

Clearly, when $C\leq \frac{\left(\left(-2S-2c_{n}\right)\rho +2S-2c_{n}\right)\beta +\alpha \left(\rho +2\right)}{2\beta \left(\rho +1\right)}$, $h^{H^{*}}>h^{T^{*}}\geq h^{Z^{*}}$; when $\frac{\left(\left(-2S-2c_{n}\right)\rho +2S-2c_{n}\right)\beta +\alpha \left(\rho +2\right)}{2\beta \left(\rho +1\right)}<C\leq \frac{\left(\left(-8S-8c_{n}\right)\rho +8S-8c_{n}\right)\beta +\left(-\beta_{1}S+4\alpha \right)\rho +\beta_{1}S+8\alpha }{8\beta \left(\rho +1\right)}$, $h^{H^{*}}\geq h^{Z^{*}}>h^{T^{*}}$; when $C>\frac{\left(\left(-8S-8c_{n}\right)\rho +8S-8c_{n}\right)\beta +\left(-\beta_{1}S+4\alpha \right)\rho +\beta_{1}S+8\alpha }{8\beta \left(\rho +1\right)}$, $h^{Z^{*}}>h^{H^{*}}>h^{T^{*}}$. Proposition 2 can thus be proven.□

**Proof of Proposition 3.** $w^{H^{*}}-w^{Z^{*}}=\frac{8((1-\rho )(C+S+c_{n})-2(C+c_{n}))\beta +4S\beta +\beta_{1}S(1-\rho )+8\alpha }{8\beta }$. If $C\leq \frac{\left(\left(-8S-8c_{n}\right)\rho +12S-8c_{n}\right)\beta -{S\rho \beta }_{1}+\beta_{1}S+8\alpha }{8\beta \left(\rho +1\right)}$, $w^{H^{*}}\geq w^{Z^{*}}$; otherwise $w^{H^{*}}<w^{Z^{*}}$. Proposition 3 can thus be proven.□

**Proof of Proposition 4.** $\frac{\partial B^{T*}}{\partial V}=\frac{\partial B^{Z*}}{\partial V}=\frac{\partial B^{H*}}{\partial V}=2>0$, $\frac{\partial A^{T^{*}}}{\partial V}=\frac{\partial A^{Z^{*}}}{\partial V}=\frac{\partial A^{H^{*}}}{\partial V}=1>0$; Proposition 4 can thus be proven.□

**Proof of Proposition 5.** $P_{M}^{H}-P_{E}^{H}=\frac{S}{4}>0$, $P_{M}^{T}-P_{E}^{Z}=S>0$, $P_{M}^{H}-P_{M}^{T}=\frac{S\beta_{1}}{8\beta }>0$, $P_{E}^{H}-P_{E}^{Z}=\frac{S(6\beta +\beta_{1})}{8\beta }>0$, $P_{E}^{H}-P_{M}^{T}=\frac{S(\beta_{1}-2\beta )}{8\beta }>0$, $\frac{\partial P_{M}^{T^{*}}}{\partial C}=\frac{\partial P_{E}^{Z^{*}}}{\partial C}=\frac{\partial P_{M}^{H^{*}}}{\partial C}=\frac{\partial P_{E}^{H^{*}}}{\partial C}=1>0$; Proposition 5 can thus be proven.□

**Proof of Proposition 6.** $\prod_{G}^{H^{*}}-\prod_{G}^{T^{*}}=\frac{S((C+\frac{11}{4}S+c_{n})\beta +\frac{\beta_{1}S}{16}-\alpha )\beta_{1}}{8\beta }$; if $0<C\leq \frac{\left(-44S-16c_{n}\right)\beta -\beta_{1}S+16\alpha }{16\beta }$, $\prod_{G}^{H^{*}}\leq \prod_{G}^{T^{*}}$; otherwise, $\prod_{G}^{H^{*}}>\prod_{G}^{T^{*}}$. The same can be proven: if $0<C\leq \frac{\left(-64S-128c_{n}\right)\beta^{2}+\left(\left(-44S-16c_{n}\right)\beta_{1}+128\alpha \right)\beta -{S\beta }_{1}^{2}+{16\alpha \beta }_{1}}{{128\beta }^{2}+{16\beta }_{1}\beta }$, $\prod_{G}^{H^{*}}\leq \prod_{G}^{Z^{*}}$; otherwise, $\prod_{G}^{H^{*}}>\prod_{G}^{Z^{*}}$. If $0<C\leq \frac{\left(-S-2c_{n}\right)\beta +2\alpha }{2\beta }$, $\prod_{G}^{T^{*}}\leq \prod_{G}^{Z^{*}}$; otherwise, $\prod_{G}^{T^{*}}>\prod_{G}^{Z^{*}}$. $\frac{\left(-44S-16c_{n}\right)\beta -\beta_{1}S+16\alpha }{16\beta }<\frac{\left(-64S-128c_{n}\right)\beta^{2}+\left(\left(-44S-16c_{n}\right)\beta_{1}+128\alpha \right)\beta -{S\beta }_{1}^{2}+{16\alpha \beta }_{1}}{{128\beta }^{2}+{16\beta }_{1}\beta }<\frac{\left(-S-2c_{n}\right)\beta +2\alpha }{2\beta }$ In conclusion, Proposition 6 is proven.□

**Proof of Proposition 7.** The proof process is similar to that of Statement 6.□

**Proof of Proposition 8.** $\frac{\partial \Pi_{M}^{T^{*}}}{\partial \;V}=\frac{4\beta \left(-C_{d}+C_{n}-C_{r}-S_{0}+V\right)\delta^{2}+4Q_{0}\beta \delta }{\delta \beta }>0$. The same can be proven: $\frac{\partial \Pi_{E}^{T^{*}}}{\partial \;V}>0$, $\frac{\partial \Pi_{G}^{T^{*}}}{\partial \;V}>0$, $\frac{\partial X_{}^{T^{*}}}{\partial \;V}>0$. The proof in the other modes is similar.□
